# A Synthetic Analogue of Neopeltolide, 8,9-Dehydroneopeltolide, Is a Potent Anti-Austerity Agent against Starved Tumor Cells

**DOI:** 10.3390/md15100320

**Published:** 2017-10-20

**Authors:** Haruhiko Fuwa, Mizuho Sato

**Affiliations:** 1 Department of Applied Chemistry, Faculty of Science and Engineering, Chuo University, 1-13-27 Kasuga, Bunkyo-ku, Tokyo 112-8551, Japan; 2 Graduate School of Life Sciences, Tohoku University, 2-1-1 Katahira, Aoba-ku, Sendai 980-8577, Japan; mizuho1207@gmail.com

**Keywords:** macrolide, cytotoxicity, necrosis, mitochondrial inhibitor, anti-austerity, autophagy, cellular energy

## Abstract

Neopeltolide, an antiproliferative marine macrolide, is known to specifically inhibit complex III of the mitochondrial electron transport chain (mETC). However, details of the biological mode-of-action(s) remain largely unknown. This work demonstrates potent cytotoxic activity of synthetic neopeltolide analogue, 8,9-dehydroneopeltolide (8,9-DNP), against starved human pancreatic adenocarcinoma PANC-1 cells and human non-small cell lung adenocarcinoma A549 cells. 8,9-DNP induced rapid dissipation of the mitochondrial membrane potential and depletion of intracellular ATP level in nutrient-deprived medium. Meanwhile, in spite of mTOR inhibition under starvation conditions, impairment of cytoprotective autophagy was observed as the lipidation of LC3-I to form LC3-II and the degradation of p62 were suppressed. Consequently, cells were severely deprived of energy sources and underwent necrotic cell death. The autophagic flux inhibited by 8,9-DNP could be restored by glucose, and this eventually rescued cells from necrotic death. Thus, 8,9-DNP is a potent anti-austerity agent that impairs mitochondrial ATP synthesis and cytoprotective autophagy in starved tumor cells.

## 1. Introduction

In normal tissues, cells are supplied with sufficient amounts of oxygen and nutrients to maintain homeostasis. In contrast, cells in tumor microenvironment often suffer from hypoxia and/or low nutrition conditions because of disordered, insufficient vascular network and excessive energy demand of rapidly proliferating cells [1,2]. Remarkably, for example, pancreatic tumor cells acquire the ability to tolerate such severe metabolic conditions; more than 50% of human pancreatic adenocarcinoma PANC-1 cells survived for 48 h in a medium completely deprived of glucose, amino acids, and serum [3]. Meanwhile, normal human fibroblasts and well-differentiated human gastric and liver cancer cells could not survive under the same culture conditions. The tolerability of tumor cells against nutrient deficiency may correlate inversely with their differentiation level. It has been reported that autophagy, a catabolic process that self-digests cytoplasmic components and recycles amino acids as energy source [4,5,6], plays an indispensable role in sustaining survival of tumor cells under nutrient deficient conditions [7,8]. Knockdown of Atg7, an autophagy related gene (Atg), by RNA interference significantly increases apoptosis in starved tumor cells [7].

It is conceivable that compounds that selectively show cytotoxicity against starved cells in tumor microenvironment, i.e., anti-austerity agents, represent potential chemotherapeutics for the treatment and cure of cancer [9]. Importantly, natural products have been a rich source of anti-austerity agents [10,11,12].

**Figure 1:**
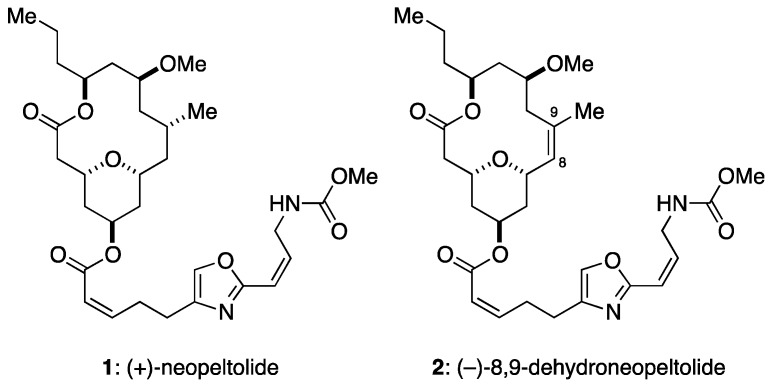
Structures of neopeltolide (**1**) and its synthetic analogue, 8,9-dehydroneopeltolide (**2**).

Wright and co-workers isolated a marine macrolide neopeltolide (**1**, Figure 1) from a deep-water sponge that belongs to the Neopeltidae family [13]. This natural product has been an intriguing synthetic target for organic chemists because of its complex structure and potent biological activity [14,15]. According to Wright et al. [13], this natural product potently inhibited in vitro proliferation of human non-small cell lung adenocarcinoma A549 cells, human ovarian sarcoma NCI-ADR-RES cells, and murine leukemia P388 cells at nanomolar concentrations, whereas it showed cytostatic activity against human pancreatic adenocarcinoma PANC-1 cells and colorectal adenocarcinoma DLD-1 cells. In addition, neopeltolide inhibited the growth of the fungal pathogen *Candida albicans* with a minimum inhibitory concentration of 0.625 μg/mL. Subsequently, Kozmin and co-workers determined that neopeltolide is a potent and specific inhibitor of complex III of the mitochondrial electron transport chain (mETC), on the basis of their finding that the growth inhibitory activity of neopeltolide in yeast cells was substantially enhanced by replacing glucose with galactose or glycerol [16]. Our group has been working on the synthesis and structure–activity relationship studies on neopeltolide and its analogues [17,18,19,20,21] and has previously reported that 8,9-dehydroneopeltolide (**2**: 8,9-DNP), a synthetic equipotent analogue of neopeltolide, induced apoptosis in human promyelocytic leukemia HL-60 cells in glucose-deprived medium [22]. However, the biological mode-of-action(s) by which neopeltolide exerts its anti-proliferative activity in human cancer cells remains largely unclear.

Here we report that 8,9-DNP showed preferential cytotoxic activity in starved tumor cells. 8,9-DNP dissipated the mitochondrial membrane potential in starved cells, resulting in suppression of mitochondrial oxidative phosphorylation and rapid decrease of intracellular ATP concentration. Impairment of cytoprotective autophagy also occurred due to the inability of cells to lipidate LC3-I to form LC3-II. Consequently, cells were severely deprived from energy sources and underwent necrotic cell death.

## 2. Results

### 2.1. 8,9-DNP Shows Prefential Cytotoxicity in Starved Tumor Cells

**Figure 2:**
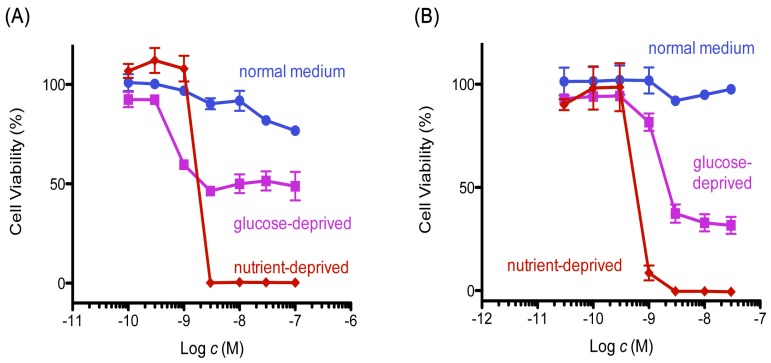
Cytotoxicity of 8,9-DNP in starved tumor cells. Cell viability was evaluated by WST-8 assay: (**A**) PANC-1 cells were incubated with various concentrations of 8,9-DNP for 24 h in nutrient-rich RPMI 1640 medium, glucose-deprived RPMI 1640 medium or NDM (*n* = 3); and (**B**) A549 cells were incubated with various concentrations of 8,9-DNP for 24 h in nutrient-rich RPMI 1640 medium, glucose-deprived RPMI 1640 medium, or NDM (*n* = 3).

Mitochondrial inhibitors have been reported to show preferential cytotoxicity and induce apoptotic death in starved PANC-1 cells [23]. Initially, we examined the cytotoxic activity of 8,9-DNP in tumor cells under normal and nutrient-starved conditions, according to the procedure described by Esumi et al. [3] (Figure 2). The cell viability did not change significantly when cells were treated with different concentrations of 8,9-DNP in nutrient-rich RPMI 1640 medium containing 10% fetal bovine serum for 24 h. In contrast, in nutrient-deprived medium (NDM), 8,9-DNP showed potent cytotoxic activity at a single-digit nanomolar concentration.

**Figure 3:**
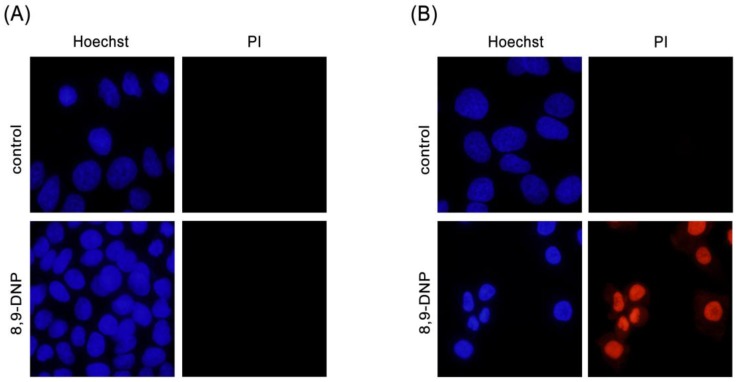
Hoechst 33342/propidium iodide (PI) double staining assay. Cells were observed with a fluorescence microscope (40× objective): (**A**) A549 cells in RPMI 1640 medium was incubated in the absence or presence of 8,9-DNP (100 nM) for 24 h and stained with Hoechst 33342/PI (*n* = 2); and (**B**) A549 cells in NDM was incubated in the absence or presence of 8,9-DNP (100 nM) for 24 h and stained with Hoechst 33342/PI (*n* = 2).

**Figure 4:**
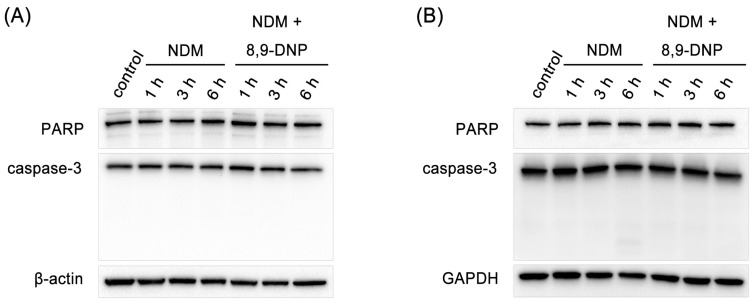
Immunoblot analysis on effect of 8,9-DNP on expression of PARP and caspase-3 in starved tumor cells: (**A**) PANC-1 cells were incubated with 8,9-DNP (100 nM) in NDM for 1, 3, or 6 h, and cell extracts were probed for indicated proteins. Control cells were grown in RPMI 1640 medium without 8,9-DNP (*n* = 3); and (**B**) A549 cells were incubated with 8,9-DNP (100 nM) in NDM for 1, 3, or 6 h, and cell extracts were probed for indicated proteins. Control cells were grown in RPMI 1640 medium without 8,9-DNP (*n* = 3).

Next, we examined by Hoechst 33342/propidium iodide (PI) double staining assay which type of cell death 8,9-DNP is induced in starved A549 cells (Figure 3). The nuclei of cells cultured in NDM for 24 h in the absence of 8,9-DNP did not show morphological change and were not stained with PI, indicating that cells survived nutrient starvation. Meanwhile, cells treated with 8,9-DNP in NDM for 24 h uniformly showed significant shrinkage of the nucleus and positively stained with PI. Cells with apoptotic morphological changes were not observed. We also examined, by immunoblot analysis, whether the apoptosis machinery is operative in starved cells. However, cleavage of neither poly-ADP ribose polymerase (PARP) nor pro-caspase-3 was observed in cells treated with 8,9-DNP, incubated in NDM (Figure 4). All these results indicated that 8,9-DNP triggered necrotic death in starved cells.

### 2.2. 8,9-DNP Dissipates the Mitochondrial Membrane Potential and Depletes Intracellular ATP Level in Starved Cells

**Figure 5:**
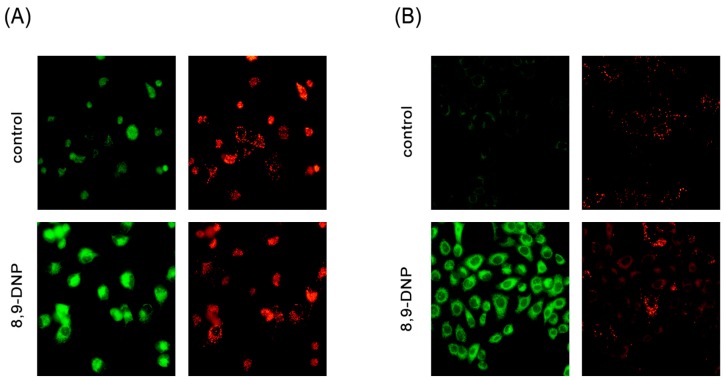
Mitochondrial membrane potential of starved tumor cells. Cells were observed with a fluorescence microscope (20× objective): (**A**) PANC-1 cells were incubated in NDM in the absence or presence of 8,9-DNP (100 nM) for 1 h and stained with JC-1 (*n* = 2). The green/red fluorescence ratio was 0.55 and 2.1 for control and treated cells, respectively; and (**B**) A549 cells were incubated in NDM in the absence or presence of 8,9-DNP (100 nM) for 1 h and stained with JC-1 (*n* = 2). The green/red fluorescence ratio was 0.52 and 6.7 for control and treated cells, respectively.

We evaluated whether 8,9-DNP inhibits mETC in starved cells by JC-1 assay [24]. This dye emits green fluorescence when it exists as a monomeric form under low concentration conditions. Once it accumulates to the mitochondrial membrane on sensing negative membrane potential, it forms J-aggregates and emits red fluorescence due to a large shift in the absorption and emission maxima. As shown in Figure 5, it was apparent that green fluorescence was intensely observed in cells treated with 8,9-DNP in NDM for 1 h when compared to control cells. This result showed that 8,9-DNP rapidly dissipated the mitochondrial membrane potential in starved cells.

**Figure 6:**
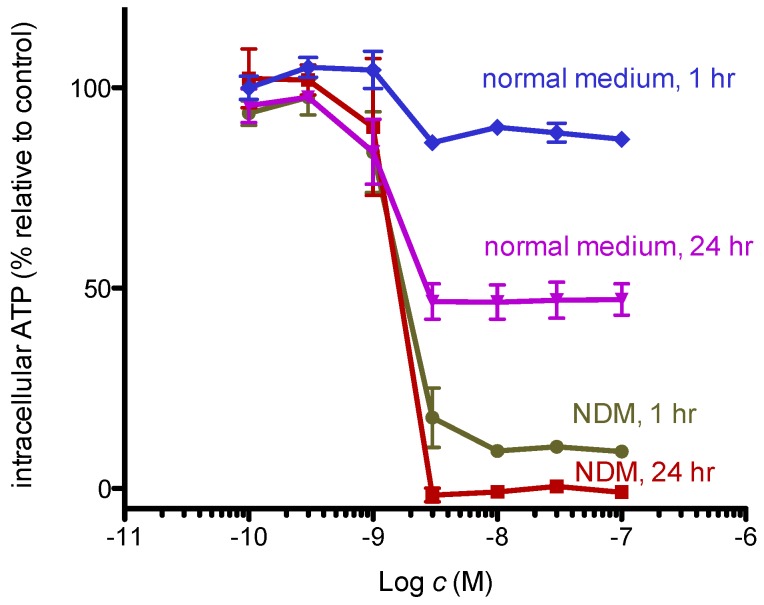
Intracellular ATP concentration of starved PANC-1 cells. Cells were incubated with various concentrations of 8,9-DNP in RPMI 1640 medium or NDM for indicated time and assayed using CellTiter-Glo^®^ assay kit. Control cells were incubated in indicated medium without 8,9-DNP for each condition (*n* = 3).

Dissipation of the mitochondrial membrane potential indicates suppression of oxidative phosphorylation. As cells in nutrient-deprived medium cannot synthesize ATP via glycolysis, they are likely dependent on mitochondrial oxidative phosphorylation. Thus, we examined whether 8,9-DNP brings about severe intracellular ATP depletion in starved PANC-1 cells by CellTiter-Glo^®^ assay (Figure 6). The intracellular ATP level of cells in nutrient-rich RPMI 1640 medium for 1 h did not change, whereas that of cells in NDM for 1 h showed significant drop to approximately 10%. This result implies that cells treated with 8,9-DNP in NDM immediately encounter energy shortage because they were unable to produce ATP via glycolysis and mitochondrial respiration. After 24 h, almost complete depletion of the intracellular ATP level was observed for cells in NDM, in line with the result of the above WST-8 assay [25].

### 2.3. 8,9-DNP Inhibited Autophagic Flux in Starved Cells

In response to nutrient withdrawal, cells activate autophagy, a catabolic process that involves non-selective degradation of cytoplasmic macromolecules and cellular organelles through a lysosomal pathway to generate amino acids that are used for protein synthesis to adapt starvation [26,27].

AMPK, a sensor of the cellular energy status, is activated by phosphorylation of a threonine residue on its α subunit on decrease in ATP/AMP ratio. AMPK induces autophagy by multiple mechanisms, including suppression of the activity of mammalian target-of-rapamycin complex 1 (mTORC1), a serine/threonine kinase [28]. Upon stimulation of autophagy, an isolation membrane appears and engulfs cytoplasmic macromolecules and cellular organelles to form an autophagosome. Conjugation of microtubule-associated protein 1 light chain 3 (LC3-I) to phosphatidylethanolamine (PE) to form LC3-II is required for maturation of the autophagosome [29]. The autophagosome then fuse with the lysosome to form an autolysosome, in which the luminal contents are finally degraded by lysosomal proteases to provide amino acids and other nutrients.

**Figure 7:**
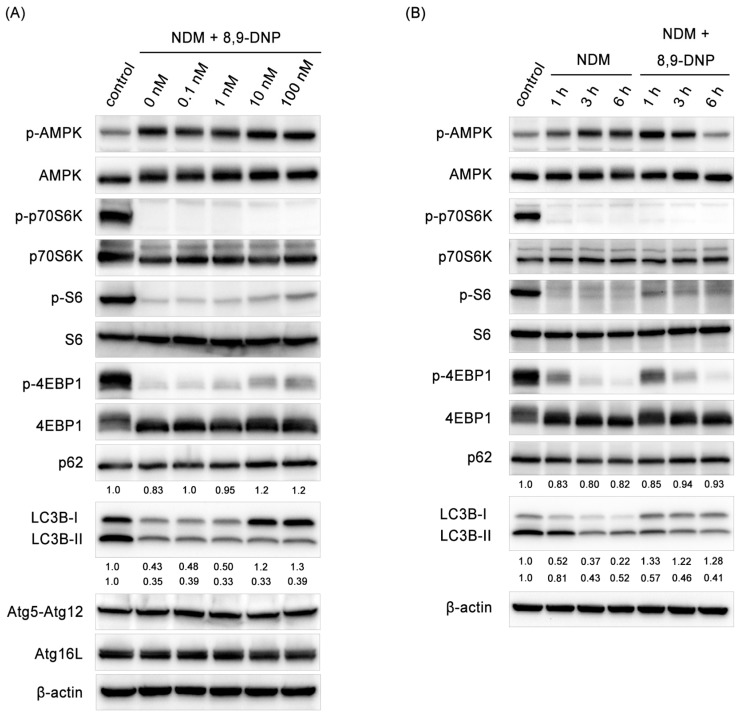
Immunoblot analysis on effect of 8,9-DNP on p-AMPK, p-p70S6K, p-S6, p-4EBP1, p62, LC3, Atg12–Atg5, and Atg16L in PANC-1 cells: (**A**) cells in NDM were treated with various concentrations of 8,9-DNP for 3 h, and cell extracts were probed for indicated proteins. Control cells were grown in RPMI1640 medium without 8,9-DNP (*n* = 3); and (**B**) cells in NDM were treated with 8,9-DNP (100 nM) for 1, 3, or 6 h, and cell extracts were probed for indicated proteins (*n* = 3). Control cells were grown in RPMI1640 medium without 8,9-DNP.

Since autophagy is known to be indispensable for cancer cells to survive nutrient-deprived conditions, we investigated whether autophagy is stimulated in starved PANC-1 cells treated with 8,9-DNP by immunoblot experiments (Figure 7A,B). Here, the phosphorylation level of AMPK was used for the assessment of the AMPK activity. It was clear that AMPK was activated under nutrient-deprived conditions both in the absence and presence of 8,9-DNP. The kinase activity of mTORC1 was evaluated by determining phosphorylation of its substrates p70S6 kinase (p70S6K), S6 kinase (S6), and eIF4E-binding protein 1 (4EBP1). Phosphorylated p70S6K (p-p70S6K) was not detected in starved cells regardless of the presence of 8,9-DNP. The phosphorylation of S6 and 4EBP1 was also suppressed, although weak phosphorylation of both was observed at a high concentration (100 nM) of 8,9-DNP. These results suggested that 8,9-DNP did not interfere with cellular energy sensing signals.

**Figure 8:**
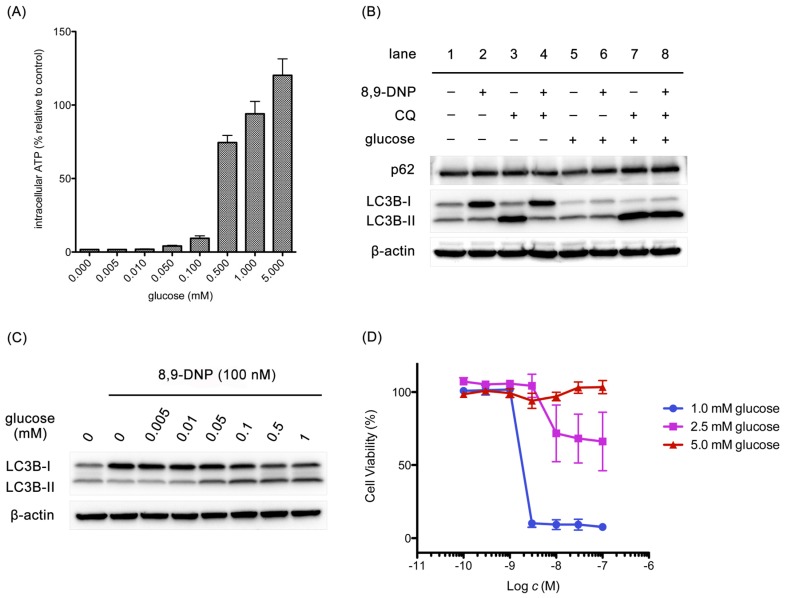
Effect of glucose on intracellular ATP concentration, expression of p62 and LC3, and cell viability: (**A**) cells were treated with 8,9-DNP (100 nM) in the presence of various concentrations of glucose in NDM for 3 h and their intracellular ATP concentration was assayed using CellTiter Glo^®^ assay kit. Control cells were incubated in NDM without 8,9-DNP (*n* = 3); (**B**) cells were treated with 8,9-DNP (100 nM) and/or chloroquine (CQ, 50 µM) in NDM (lanes 1–4) or in NDM + 5 mM glucose (lanes 5–8), and cell extracts were probed for indicated proteins (*n* = 3); (**C**) cells were treated with 8,9-DNP (100 nM) in the presence of various concentrations of glucose in NDM for 3 h, and cell extracts were probed for indicated proteins. Control cells were incubated in NDM without 8,9-DNP (*n* = 3); and (**D**) cells were treated with various concentrations of 8,9-DNP in the presence of 1.0, 2.5, or 5.0 mM glucose in NDM for 24 h and their viability was evaluated using WST-8 assay (*n* = 3).

However, it was found that 8,9-DNP inactivated autophagy on the basis of the findings that the conversion of LC3-I to its lipidated counterpart LC3-II was suppressed and that the expression level of p62, a polyubiquitin-binding protein, increased dose-dependently [30,31,32]. An autophagic flux assay was further performed using chloroquine as a lysosomal inhibitor to exclude the possibility that the low expression level of LC3-II was due to its rapid degradation by the lysosome (Figure 8B) [32]. A similar set of results was obtained when starved A549 cells were exposed to 8,9-DNP (Supplementary Figure S1).

Among a number of autophagy related genes (Atg), Atg12–Atg5–Atg16L complex is required for the formation of autophagosomes [33,34]. It has been reported that Atg5-silenced PANC-1 cells are more prone to undergo cell death under nutrient-deprived conditions [35]. Thus, it was examined whether the expression level of Atg12–Atg5 conjugate and Atg16L declines in the presence of 8,9-DNP. However, neither Atg12–Atg5 conjugate nor Atg16L was affected with 8,9-DNP.

### 2.4. Glucose Restores Autophagic Flux and Rescues Starved Cells

It is known that several steps in the autophagic machinery, including lipidation of LC3-I to form LC3-II, are dependent on ATP [36]. At this point, it was questioned if the impairment of autophagy could be attributed to rapid, severe depletion of intracellular ATP concentration as a consequence of mETC inhibition by 8,9-DNP. Accordingly, it was examined whether glucose restores intracellular ATP level to stimulate autophagy in starved cells in the presence of 8,9-DNP (Figure 8). After a three-hour incubation, the intracellular ATP level was increased by glucose in a dose-dependent manner (Figure 8A). Immunoblot experiments at the same time point showed that, in the absence of glucose, 8,9-DNP compromised lipidation of LC3 (Figure 8B, lanes 1–4), whereas, in the presence of 5 mM glucose, 8,9-DNP had no significant effect on the autophagic flux (Figure 8B, lanes 5–8). It was also confirmed that glucose promoted the conversion of LC3-I to LC3-II in a dose-dependent fashion (Figure 8C). These results suggested that cells utilized glucose as an energy source for stimulating cytoprotective autophagy. Notably, the concentration of glucose required for stimulating autophagy coincided with that required for increasing intracellular ATP level. Furthermore, glucose dose-dependently rescued starved cells from necrotic death induced by 8,9-DNP, as demonstrated by WST-8 assay after 24 h (Figure 8D). It appears that 1 mM glucose, approximating the glucose concentration in tumor microenvironment, [37] was sufficient for cells to induce cytoprotective autophagy at 3 h. Nevertheless, most of the cells underwent death after 24 h of incubation with nanomolar concentrations of 8,9-DNP probably because of the eventual shortage of energy. Meanwhile, cells in medium containing 5 mM glucose did not show any drop in viability even in the presence of submicromolar concentrations of 8,9-DNP; it is likely that the cells were able to produce sufficient amounts of ATP by stimulating glycolysis and autophagy under these conditions.

## 3. Discussion

Solid tumors often have regions that chronically or transiently suffer from low oxygen and/or nutrient supplies because of rapid proliferation of tumor cells and insufficient angiogenesis. In particular, pancreatic cancer is one of the most aggressive malignancies although pancreatic cancer tissues have poor blood supply due to hypovascularity. Tumor cells in cancer tissues have remarkable ability to adapt to unfavorable tumor microenvironment [2,3]. Apoptosis-defective tumor cells stimulate cytoprotective autophagy to secure amino acids as an energy source for short-term survival under metabolic stress conditions [7,8]. Actually, it has been shown that cells treated with Atg5 siRNA results in increased cell death under nutrient-deprived conditions, compared to those treated with a scrambled siRNA [35]. However, long-term induction of autophagy may result in autophagic cell death with excessive cytoplasmic vacuolization. Simultaneous impairment of apoptosis and autophagy in tumor cells promotes necrosis, although this mode of cell death in vivo is normally associated with inflammation and may result in tumor growth [38].

Given the marked survival ability of tumor cells, anti-austerity agents, compounds that cancel the ability of tumor cells to tolerate nutrient deficiency, are of significant interest [9,10,11,12]. Wortmannin and chloroquine, autophagy inhibitors, activate caspase-dependent apoptosis in starved PANC-1 cells [35]. AG1024 and I-OMe-AG538, insulin-like growth factor-1 (IGF-1) receptor tyrosine kinase inhibitors, show selective cytotoxicity in starved pancreatic tumor cells to induce necrotic death [39]. Grandifloracin, a natural product isolated from the stem of *Uvaria dac*, sensitizes PANC-1 cells to autophagic cell death under nutrient-deprived conditions [40]. Naturally-occurring mitochondrial inhibitors are shown to be preferential cytotoxic agents against starved PANC-1 cells [23]. Using annexin V/PI double staining and flow cytometric analysis, Momose and co-workers reported that mitochondrial inhibitors, efrapeptin F (complex V inhibitor), rotenone (complex I inhibitor), atpenin A5 (complex II inhibitor), and antimycin A (complex III inhibitor), significantly increase the population of early- and late-apoptotic cells. On the basis of these reports, it appears that several mechanisms are available for selective targeting of starved tumor cells.

The present study demonstrated that 8,9-DNP, a synthetic analogue of neopeltolide, exerted selective cytotoxicity in starved tumor cells. 8,9-DNP induced rapid dissipation of the mitochondrial membrane potential, depletion of intracellular ATP and, finally, necrotic cell death. Significant loss of cellular energy would be attributable to the unavailability of glucose with simultaneous inhibition of mETC. Although tumor cells are competent to obtain amino acids by stimulating autophagy under nutrient-deprived conditions, 8,9-DNP severely depleted intracellular ATP within a short time and consequently prohibited the autophagic machinery to operate. Specifically, in this case, the lipidation of LC3-I to form LC3-II was suppressed, possibly because this process requires adenylation of LC3-I with Atg7 in an ATP-dependent manner [36]. Accordingly, starved cells underwent necrotic cell death because of their inability to produce cellular energy. Mitochondrial ATP synthesis should be the lifeline of tumor cells under nutrient deficiency.

The type of cell death induced by anti-austerity agents depends on their mechanism-of-action. It is known that nutrient starvation triggers cell cycle arrest and apoptosis in tumor cells. In fact, autophagy inhibitors have been shown to activate the caspase cascade to induce apoptosis in starved PANC-1 cells [35]. Autophagic cell death should also be possible as a result of excessive stimulation of autophagy under starvation conditions [41]. Meanwhile, it is also known that execution of apoptosis or autophagy requires a certain level of intracellular ATP concentration [42]. It is speculated that the apoptosis-inducing ability of 8,9-DNP in human promyelocytic leukemia HL-60 cells under glucose-deprived conditions, described in our previous work [22], would be ascribable to the availability of nutrients other than glucose, which provide cells with sufficient amounts of ATP to activate the apoptosis machinery. Anti-austerity agents that target mETC likely cause immediate shortage of cellular energy and, eventually, necrotic cell death in tumor cells under extreme starvation conditions.

Withdrawal of nutrients, such as glucose, amino acids, and serum, has been commonly considered a stimulus for autophagy induction in cultured cells. As shown in Figure 8B, it was observed for PANC-1 cells that autophagy could be stimulated in NDM with or without glucose. However, the autophagic flux was estimated to be approximately 1.2-fold higher for cells in NDM *with* glucose than those in NDM without glucose. Moreover, in the presence of 8,9-DNP, it was found that glucose was necessary for maintaining autophagic flux and rescuing cells from necrotic death. Therefore, it can be considered that glucose is a stimulator, rather than an inhibitor, of autophagy in starved PANC-1 cells. Previous study has described that glucose induces autophagy in NIH 3T3 cells under starvation conditions and that the induction of autophagy is independent of AMPK and mTOR and mainly relies on the activation of p38 mitogen-activated protein kinase (MAPK) [43]. In line with previous study, it was found that 8,9-DNP repressed the activity of p38 MAPK in the absence of glucose, but that glucose promoted the phosphorylation of p38 MAPK independently of 8,9-DNP (data not shown). Nevertheless, there is a controversial report describing that p38 MAPK is a negative regulator of autophagy [44]. The mechanism by which p38 MAPK regulates autophagy in starved tumor cells may need further investigation [45].

## 4. Materials and Methods

### 4.1. Materials

The human lung adenocarcinoma A549 cell line and the human pancreatic adenocarcinoma PANC-1 cell line were provided from RIKEN BRC (Tsukuba, Japan) through the National Bio-Resource Project of the MEXT, Japan. Primary antibodies were obtained and diluted with 5% Blocking One (Nacalai Tesque, Kyoto, Japan) for use as follows: rabbit anti-caspase-3 monoclonal antibody (1:1000), rabbit anti-PARP monoclonal antibody (1:1000), rabbit anti-p-AMPK monoclonal antibody (1:1000), rabbit anti-AMPK monoclonal antibody (1:1000), rabbit anti-p-p70S6K monoclonal antibody (1:1000), rabbit anti-p70S6K monoclonal antibody (1:1000), rabbit anti-p-S6 monoclonal antibody (1:2000), rabbit anti-p-4EBP1 monoclonal antibody (1:1000), rabbit anti-4EBP1 monoclonal antibody (1:1000), and rabbit anti-LC3B monoclonal antibody (1:1000) from Cell Signaling Technology; mouse anti-S6 monoclonal antibody (1:200) and mouse anti-β-actin monoclonal antibody (1:500) from Santa Cruz Biotechnology; rabbit anti-p62 polyclonal antibody (1:1000), mouse anti-Atg12–Atg5 monoclonal antibody (1:1000), mouse anti-Atg16L monoclonal antibody (1:1000), and HRP-conjugated mouse anti-GAPDH monoclonal antibody from Medical and Biological Laboratories. 8,9-Dehydroneopeltolide (8,9-DNP) was synthesized and purified as described previously [20] and purified by preparative high-performance liquid chromatography. RPMI 1640 medium used in this study was purchased from Nacalai Tesque, supplemented with 10% fetal bovine serum, 100 units/mL of penicillin, and 100 µg/mL of streptomycin, and filter-sterilized before use. Nutrient-deprived medium (NDM) was prepared according to the procedure described by Esumi et al. [3] The composition of NDM was as follows: CaCl_2_·2H_2_O 265 mg/L, FeNO_3_·9H_2_O 0.1 mg/L, KCl 400 mg/L, MgSO_4_·7H_2_O 200 mg/L, NaCl 6400 mg/L, NaHCO_3_ 700 mg/L, NaH_2_PO_4_ 125 mg/L, phenol red 15 mg/L, HEPES buffer (pH 7.4) 25 mmol/L, and MEM vitamin solution (Thermo Fisher Scientific, Waltham, MA, USA).

### 4.2. Cell Viability Assay

Cells were cultured for 2–3 days in RPMI 1640 medium maintaining under a 5% CO_2_/air atmosphere in a CO_2_ incubator at 37 °C. Exponentially growing cells were trypsinized, harvested, and re-suspended in RPMI 1640 medium at a density of 1.0 × 10^5^ cells/mL. The cell suspension (100 µL) was distributed to wells of a 96-well microtiter plate and incubated in a CO_2_ incubator at 37 °C for 16–24 h. The cells were rinsed with D-PBS and placed with appropriate medium (100 µL). The cells were then treated with various concentrations of 8,9-DNP (1 µL) and incubated in a CO_2_ incubator at 37 °C for 24 h. The cells were rinsed with D-PBS and placed with RPMI 1640 medium containing 5% WST-8. After incubation in a CO_2_ incubator at 37 °C for additional 5 h, the absorbance at 450 nm was measured for each well by a microplate reader (Synergy^®^HT Multi-Mode Microplate Reader, BioTek Instruments, Winooski, VT, USA). The relative absorbance values were plotted against sample concentrations (the relative absorbance values for 0% and 100% cell viability were obtained with blank and 10% MeOH/H_2_O, respectively).

### 4.3. Nuclear Morphology Analysis

The Höechst 33342/propidium iodide double staining technique was used to observe nuclear morphology and to discriminate living and dead cells. Cells were incubated in the presence or absence of 8,9-DNP (100 nM) in an appropriate medium at 37 °C for 24 h. The cells were rinsed once with D-PBS and then treated with 10 µg/mL Höechst 33342 and 5 µg/mL propidium iodide at room temperature for 30 min. The cells were rinsed once with D-PBS and then observed under an inverted fluorescence microscope (IX-73, Olympus, Tokyo, Japan) equipped with a 40× objective.

### 4.4. Mitochondrial Membrane Potential Assay

The JC-1 assay technique was used to observe the mitochondrial membrane potential. After treatment of cells with 8,9-DNP (100 nM) in an appropriate medium for 1 h, the cells were rinsed with D-PBS and stained with JC-1 (5 µg/mL) at room temperature for 15 min. The cells were rinsed with D-PBS and observed under an inverted fluorescence microscope (IX-73, Olympus, Tokyo, Japan) equipped with a 20× objective. Image J software (NIH) was used for image processing and quantification of the fluorescence intensity.

### 4.5. Measurement of Intracellular ATP Concentration

Intracelluar ATP concentration of PANC-1 cells was determined by using CellTiter-Glo^®^ assay kit (Promega, Kyoto, Japan) according to the manufacturer’s protocol.

### 4.6. Preparation of Cell Extract

After treatment of cells with 8,9-DNP in nutrient-deprived medium, the cells were harvested in ice-cold D-PBS using a cell scraper. After centrifugation (500× *g*, 4 °C, 5 min), the cells were lysed by incubation on ice with 2% SDS buffer supplemented with protease inhibitor cocktail (Nacalai Tesque, Kyoto, Japan). The resultant cell extract was sonicated at 4–8 °C, diluted with equal volume of 100 mM DTT containing bromophenol blue and boiled at 95 °C for 4 min. After cooling on ice, the resultant solution was centrifuged (500× *g*, 4 °C, 2 min), and the supernatant was saved as the whole cell extract and stored at −80 °C before use.

### 4.7. Immunoblot Analysis

The whole cell extract prepared above was resolved on a 7.5% or 12% SDS-polyacrylamide gel and then transferred onto a PVDF membrane. The membrane was blocked with Blocking One (Nacalai Tesque, Kyoto, Japan) at room temperature for 30 min, rinsed with T-TBS (×3), and probed with an appropriate primary antibody at room temperature for 1 h or at 4 °C overnight. The membrane was rinsed with T-TBS (×3) and probed with an appropriate HRP-conjugated secondary antibody at room temperature for 1 h. The membrane was rinsed with T-TBS (5 min × 3) and developed with Chemi-Lumi One (Nacalai Tesque, Kyoto, Japan). Chemiluminescence was detected on a ChemiDoc XRS + Imaging System (Bio-Rad, Hercules, CA, USA). The intensity of observed bands was quantified and normalized to the loading control using Image Lab software (Bio-Rad, Hercules, CA, USA).

## Supplementary Materials

The following are available online at www.mdpi.com/1660-3397/15/10/320/s1; **Figure S1**. Immunoblot analysis on effect of 8,9-DNP on p-p70S6K, p-4EBP1, p62, and LC3 in A549 cells.
