# Supplementary material for: A Synthetic Analogue of Neopeltolide, 8,9-Dehydroneopeltolide, Is a Potent Anti-Austerity Agent against Starved Tumor Cells
Source: Mar Drugs. 2017 Oct 20;15(10):320. doi: 10.3390/md15100320 (PMC5666428; doi:10.3390/md15100320)
Supplement: Supplementary file 1 [file marinedrugs-15-00320-s001.pdf]

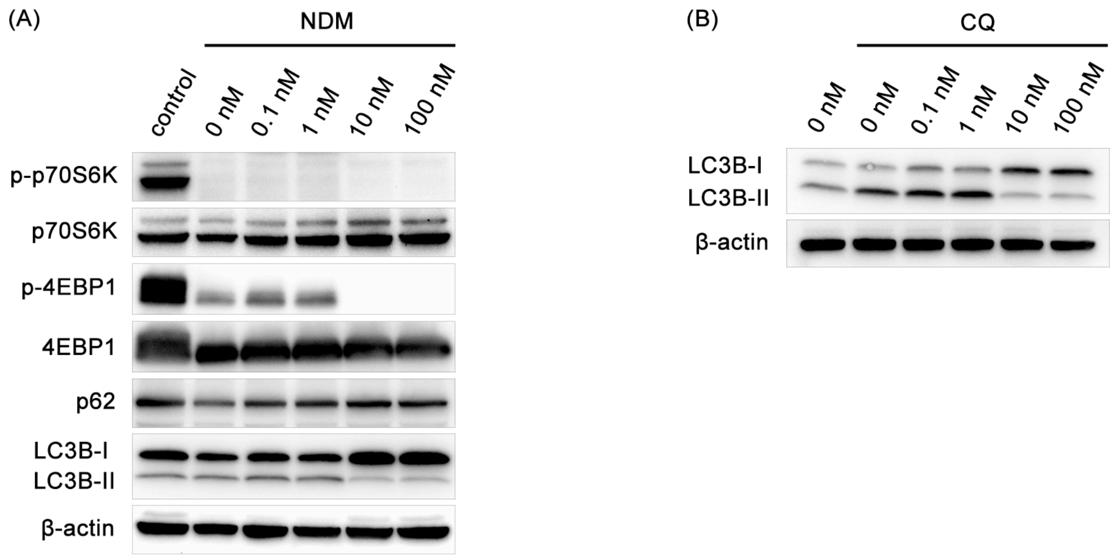

Figure S1. Immunoblot analysis on effect of 8,9-DNP on p-p70S6K, p-4EBP1, p62, and LC3 in A549 cells: (**a**) Cells in NDM were treated with various concentrations of 8,9-DNP for 3 h, and cell extracts were probed for indicated proteins; (**b**) Cells in NDM were treated with various concentrations of 8,9-DNP in the presence of chloroquine (CQ, 50 μM) for 3 h, and cell extracts were probed for indicated proteins.
